# Wear in Antagonist Teeth Produced by Monolithic Zirconia Crowns: A Systematic Review and Meta-Analysis

**DOI:** 10.3390/jcm9040997

**Published:** 2020-04-02

**Authors:** María Fernanda Solá-Ruíz, Alejandra Baima-Moscardó, Eduardo Selva-Otaolaurruchi, José María Montiel-Company, Rubén Agustín-Panadero, Carla Fons-Badal, Lucía Fernández-Estevan

**Affiliations:** Department of Dental Medicine Faculty of Medicine and Dentistry, University of Valencia, C/Gascó Oliag n1, 46010 Valencia, Spain; m.fernanda.sola@uv.es (M.F.S.-R.); eduardo.j.selva@uv.es (E.S.-O.); jose.maria.montiel@uv.es (J.M.M.-C.); ruben.agustin@uv.es (R.A.-P.); carla.fons@uv.es (C.F.-B.); lucia.fernandez-estevan@uv.es (L.F.-E.)

**Keywords:** crown, monolithic zirconia, dental wear, antagonist teeth

## Abstract

Background: The aim of this systematic review and meta-analysis was to determine the wear sustained in the natural antagonist tooth in cases of full-coverage fixed-base prosthetic restorations or monolithic zirconia tooth-supported crowns, as well as to determine the wear in the restoration itself, both in the short- and medium-term and considering the factors that may influence wear. Material and methods: A systematic literature review and meta-analysis, based on Preferred Reporting Items for Systematic Reviews and Meta-Analyses (PRISMA) recommendations, of clinical studies that evaluated wear in antagonist teeth in relation to fixed-prosthesis monolithic zirconia crowns. A total of 5 databases were consulted in the literature search: Pubmed-Medline, Cochrane, Scopus, Embase and Web of Science (WOS). After eliminating duplicated articles and applying the inclusion criteria, eight articles were selected for the qualitative analysis and four for the quantitative analysis. Results: Mean maximum wear of the antagonist tooth in relation to monolithic zirconia crowns of magnitude 95.45 µm (CI at 95% 79.57–111.33) was observed. By using a meta-regression model (R^2^ = 0.92) the significant effect of time in maximum wear rate (*p* < 0.001) was observed, estimated at 6.13 µm per month (CI at 95% 3.99–8.27). Furthermore, monolithic zirconia crowns are subject to a mean maximum wear of 58.47 µm (CI 95% 45.44–71.50). By using a meta-regression model (R^2^ = 0.53) the significant effect of time in the maximum wear value was observed (*p* = 0.053), estimated at 3.40 µm per month (CI al 95% −0.05–6.85). Conclusions: Monolithic zirconia crowns lead to a progressive maximum wear of the antagonist tooth over time which is greater than the maximum wear sustained in the crown itself. It is not possible to establish an objective and quantitative objection in relation to natural enamel wear or metal–ceramic crowns.

## 1. Introduction

Complete ceramic-based restorations have become increasingly popular as they are free of metals, are aesthetically pleasing and furthermore are biocompatible. Of noteworthy interest are those manufactured according to zirconia CAD-CAM technology. Zirconium dioxide or zirconia stabilized in the tetragonal phase (3Y-TZP) is a material that offers an opacity similar to that of metal coated with ceramic feldspars. However, a frequent problem in the long term is the propensity of veneered zirconia to chipping in the porcelain coating [1,2].

Following from this, there has been extensive development of monolithic zirconia materials milled from solid blocks using CAD-CAM technology and without any additional porcelain coating. This material offers favorable mechanical characteristics such as high degree of hardness (1387 Hv) and its resistance to fracture (900–1200 MPa) [3,4] has led to the widespread use of monolithic zirconia (MZ) as an alternative to veneered zirconia restorations, as well as to the classical metal–ceramic restorations indicated in fixed dental prostheses (FDP) [5].

The high resistance to fracturing offered by MZ allows for the deployment of crowns with minimal thicknesses of approximately 0.5 mm, thereby contributing to the biological benefit arising from the preservation of dental matter [6,7,8]. There is also the absence of chipping, hence making it an excellent restorative material [9]. However, due to their mediocre aesthetics, restorations are limited to the posterior area [10].

The possible damage arising from abrasion between MZ and the antagonist natural tooth as a result of the hardness of the material, much greater than that of porcelaneous coatings, with values varying between 481 and 647 Hv [11] and augmented by the unevenness of the restoration surface area, above all in cases of specific milling for occlusal adjustment or wear [7,10,12,13,14].

Different in vitro studies have shown that zirconia monolithic crowns cause less wear to the antagonist tooth or a degree of wear which is comparable to ceramic or ceramic–metallic restorations [15,16,17,18]. Making direct comparisons between such study is difficult as there are differences in the surface finish of the material, the type of material used and indeed the methods used to analyze the wear [13].

Notwithstanding this, intraoral wear is a complex phenomenon governed by physical, chemical and biological factors; hence, in vitro studies cannot fully simulate real-life clinical wear [1].

One of the factors that may influence the results of enamel wear on such restorations is the position of the restoration, either molars or premolars [7], as well as the degree of attrition or the parafunctional habits of patients [3,14,19]. Besides, reference is also made to the influence of the patient’s sex or age [14].

Following from this, the object of this study is to determine the wear in the antagonist tooth with respect to monolithic zirconia crowns and to the crown itself in the short- and medium-term in patients with fixed prostheses and evaluating the factors influencing the said wear.

## 2. Materials and Methods

The systematic literature review was carried out in accordance with the PRISMA recommendations (PRISMA: Preferred Reporting Items for Systematic Reviews and Meta-Analyses) [20] with prior registration in PROSPERO (Registration number CRD42019133399).

The PICO question (population, intervention, comparison, outcome) was: What is the wear sustained in the antagonist tooth with respect to monolithic zirconia restorations in the short- and medium-term for patients with fixed prosthesis?. The designation “P” (patient) refers to the type of patients studied, patients who have a fixed prosthesis. The designation “I” (intervention) gathered up the terms referring to restorative treatments with monolithic zirconia. The designation “O” (Outcome) grouped together the terms linked to the behavior of the said restorations over a given period. In this PICO question there was comparison or control group. Once the PICO question and the terms in each section were defined respectively, the said terms were subjected to Boolean operations set as ‘OR’ and ‘AND’.

In short, the search strategy was defined as:

((((((((“humans”[MonthH Terms]) OR “adult”[MonthH Terms]) OR “middle aged”[MonthH Terms]) OR “adolescent”[MonthH Terms]) OR “male”[MonthH Terms]) OR “female”[MonthH Terms])) AND (((((“monolithic zirconia”) OR “monolithic zirconia ceramics”) OR “monolithic zirconia prostheses”) OR “monolithic zirconia single crowns”) OR “monolithic zirconia restorations”)) AND (((((((“behaviour”) OR “complications”) OR “analyses, prosthesis failure”[MonthH Terms]) OR “enamel wear”) OR “enamel wear rates”) OR “fracture”) OR “clinical longevity”).

An electronic search took place in the following databases: Pubmed-Medline, Scopus, Embase, Cochrane, as well as Web of Science (WOS). The systematic review and meta-analysis span all the literature published up to October 30, 2019. Applying the following inclusion criteria: clinical trials, case-control studies and cohort studies in humans and with a sample size greater than ten crowns. No restriction was placed on language of publication. The following exclusion criteria were applied: reports on clinical cases, articles that expressed opinions and in vitro studies were excluded.

Two members of the research team (A.B.M, F.S.R) carried out duplicated database searches independently. The headings and abstracts were selected by applying inclusion and exclusion criteria. One researcher (A.B.M.) collated data for relevant variables. The systematic review was carried out by (A.B.M.) and the posterior meta-analysis was performed by a researcher not involved in the selection process (J.M.C.).

The variables registered in each of the studies were: author, year of publication, title and journal, type of study, sample size (*n* = crowns), losses of individual crowns, types of mechanical complications (occlusal wear), inclusion and exclusion criteria in the studies, results of the studies, patient follow-up time, restoration positions, treatment given to surface, wear measurement methods and quality of the studies.

The quality of the studies was independently analyzed by the same researchers. To evaluate the quality of the cohort studies the Newcastle–Ottawa Quality Evaluation Scale was used (NOS) [21]. This scale is made up of 8 items, organized in three categories: selection of patients, degree of comparability of study groups in question, and results, whereby a total score of 9 stars were obtained. High quality studies were considered as those with 3 or 4 stars in the selection domain; 1 or two stars for comparability domain and 2 or 3 stars in the outcome domain. The quality of clinical trials was judged according to the PEDro scale [22]. The scale was made up of 11 items (each one evaluated as being either present or absent) with a score ranging from 0 to 11. Studies with a score of 5 or above were deemed to be of high quality and presenting a low bias risk.

The data included in the meta-analysis have been combined according to the random effects model and expressed as forest plots. The mean difference, confidence intervals (CI) at 95%, as well as the z- and *p*-values for each individual study are supplied. The heterogeneity study was carried out by an I^2^ statistical calculation and applying Cochran’s Q-test. To explain the heterogeneity detected, a meta-regression analysis was applied according to the maximum likelihood model.

The estimated effect size is the mean of wear measured in µm produced in monolithic zirconia crowns, as well as in the natural antagonist tooth during and also at the end of the follow-up period.

In relation to the selection bias study, Funnel graphs were devised; Duval’s method and Tweedie’s trim and fill were also applied to analyze studies in case of asymmetries, thereby enabling a new estimation of effect to be obtained. Lastly, the Intercept in Egger’s lineal regression was calculated.

The reference significance level for the analyses was 5% (α = 0.05). The software used for the meta-analysis was Comprehensive Methanalyses 3.0.

## 3. Results

The initial electronic search identified 60 articles in PubMed, 74 in Embase, 110 in Scopus, 28 in Cochrane and 85 in WOS. From a total of 357 articles, 142 were ignored as they were duplicates.

**Figure 1:**
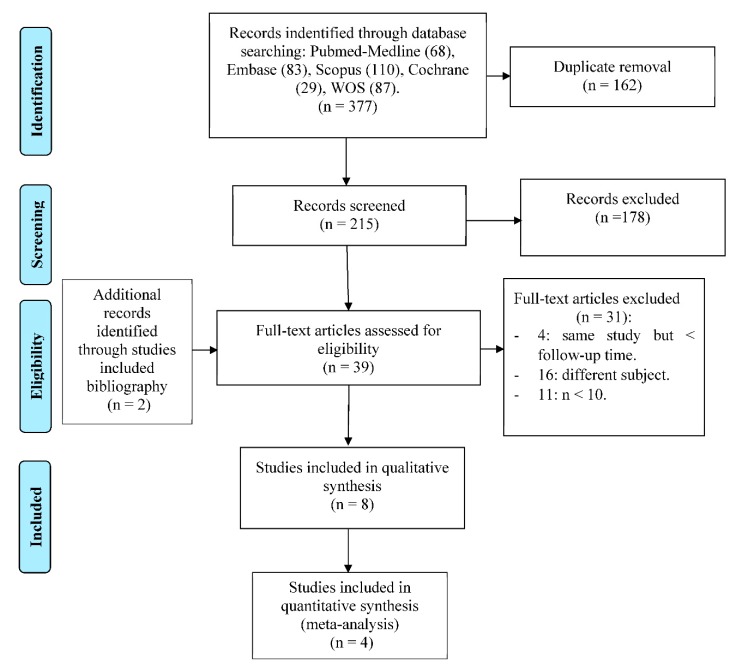
Preferred Reporting Items for Systematic Reviews and Meta-Analyses (PRISMA) Flow diagram.

After reading the titles and abstracts, a further 178 were eliminated, because they did not meet the inclusion criteria (as most of them were in vitro studies or clinical case reports) or because they exposed a different subject to our study objective, leaving a total of 37 studies. Of these, 31 were rejected for 3 reasons: having a crown sample size less than 10 (*n* < 10), addressing a different question, or being the same study published prior to another one but with a smaller sample. Of the 6 remaining studies, 2 additional articles were added by way of the bibliographies of the studies included. Finally, 8 articles were included for the qualitative synthesis and a 4 for the quantitative synthesis or meta-analysis as all the necessary data and variables were present (Figure 1). Of the 8 studies included in the systematic review, one of them is a cohort study and the remaining 7 were clinical trials. 

The evaluation of results for quality of methodology used in the Newcastle–Ottawa (NOS) and PEDro scales are shown in Table 1 and Table 2. The cohort study indicated a high quality according to the Newcastle–Ottawa Scale with a score of 6 [21] (Table 1). In relation to the clinical trials included, one study presented a low-quality evaluation [9], while the 6 remaining studies presented a high-quality evaluation according to PEDro with scores > 5 [22] (Table 2). Once again, quality was increasingly compromised due to non-compliance in the items concerned with the subject, the treatment itself or blinding in the measurement process.

In this article, the exposed cohort is the antagonist teeth to monolithic zirconia crowns. No comparison is made with a control group (natural tooth) so there is no unexposed cohort. For this reason, points 2, 5 and 6 are not applicable.

The qualitative synthesis included 8 articles (Table 3). The samples varied between 10 and 60 crowns analyzed in patients with ages ranging from 18 and 78 years.

The said studies have measured the occlusal wear or complications of the natural antagonist tooth: two of them [7,14] compared the enamel wear in the antagonist enamel in monolithic zirconia restorations in relation to the enamel wear sustained in contralateral natural teeth (control teeth); both studies indicate a greater degree of wear in the antagonist enamel relative to zirconia restorations compared to enamel wear in control teeth (*p* < 0.05). In the study by Lohbauer and Reich [10] there is measurement of the wear sustained in monolithic zirconia restorations and in the natural antagonist tooth; the results do not indicate a statistically significant difference between both types of antagonist teeth. Pathan [9] only indicates the mean wear in the natural antagonist tooth, while Hartkamp [23] states the maximum mean loss in the antagonist tooth where there have been monolithic zirconia restorations. However, in the study by Esquivel-Upshaw [13] there were no statistically significant differences between the wear in the antagonist enamel with respect to monolithic zirconia restorations and with respect to ceramic–metallic ones and in the natural tooth. Lastly, Tang [3], measured the antagonist enamel wear according to the degree of attrition, where 6.12% of monolithic zirconia restorations produce Grade 1 wear in the antagonist enamel (wear only in the enamel and changes in the occlusal surface morphology); a further 6.12% produces Grade 2 wear (light wear in the dentine, exposure of the occlusal dentine with an area ≤2 mm^2^, and a reduction in the crown height due to morphological changes on the occlusal surface).

Furthermore, Pathan [9] detected anatomical changes in the crown and in its surface texture. Kitaoka **[19]** observed the presence of fissures or cracks in the enamel of antagonist teeth in zirconia restorations in 10.34% of cases and there was attrition in 24.14% of cases.

Results in the meta-analysis have been compared using mean maximum vertical loss results (μm). All the studies analyse wear in the occlusal contact areas (Table 4), except one [10], which analyses wear in the regions of interest of the occlusal surface, corresponding to the areas where relevant facets of wear are located.

**Figure 2:**
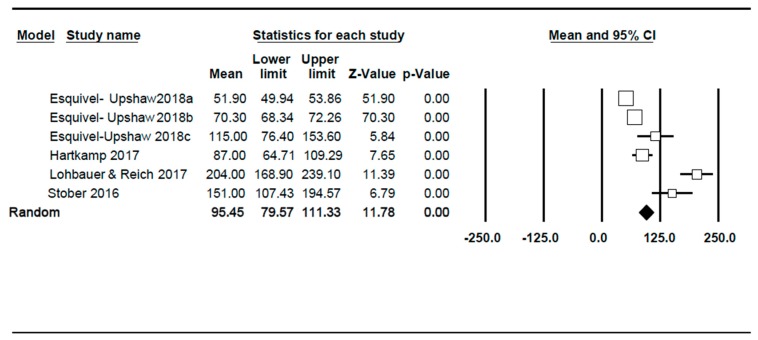
Forest-plot. The mean of maximum wear reported by each author over time and indicating the calculation of mean of global effect for the tooth antagonist to monolithic zirconia restorations.

The studies included in the meta-analysis have estimated a maximum vertical wear ranging between 51.9 µm and 204 µm in a period between 6 and 24 months for the tooth that is antagonist to monolithic zirconia crowns (Figure 2). The combination of studies based on a random effects model has indicated a mean maximum wear of 95.45 µm, with a confidence interval at 95% (79.57–111.33). The meta-analysis has revealed a high heterogeneity where I^2^ = 98.1, while Q test = 261.5 (*p* < 0.001). The funnel plot indicates some asymmetry. The main source of heterogeneity is time, as studies spanning 6- to 24-month periods are included.

**Figure 3:**
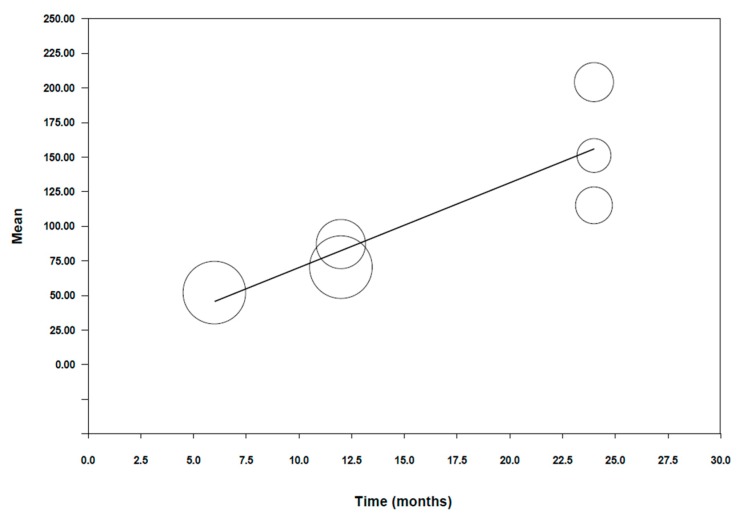
Graph depicting meta-regression of wear in the antagonist tooth vs. time.

**To explain the high degree of heterogeneity found, a meta-regression (Figure 3) was performed.** A significant model was obtained with a Q test = 31.56 (*p* < 0.001). This is indicative that the variable time is a significant variable in the model. The beta coefficient of the intercept was 8.91 with a CI at 95% (−24.02–41.83), giving a predictive capacity of 92% (R^2^ = 0.92). The beta coefficient of time was 6.13 with a *p* value < 0.001 and a CI at 95% (3.99–8.27). We can therefore conclude that there is a maximum wear of 6.13 microns per month for the tooth that is antagonist to monolithic zirconia restorations.

**Figure 4:**
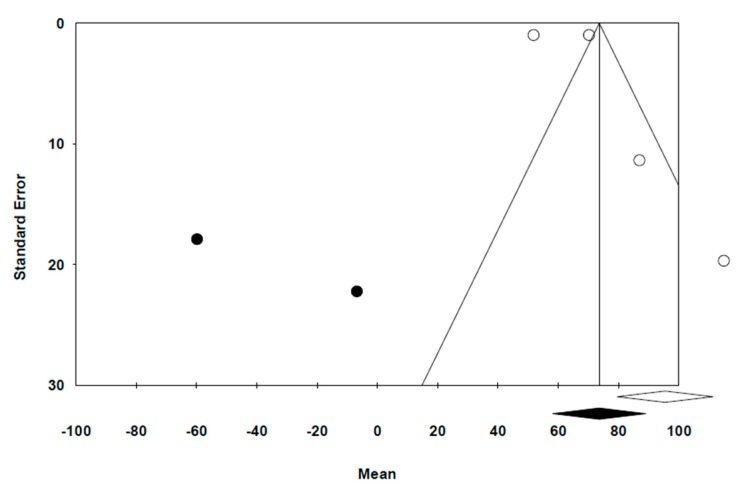
Funnel Plot with trim and fill (Duval and Tweedie).

The funnel plot (Figure 4) indicates the distribution of the results in the studies ss asymmetrical, thereby suggesting a publication bias. The *trim and fill* method has given a new estimation of effect, with a mean value of 73.6 µm, which differs from the original mean generated in the random effect method (95.45 µm), thereby maintaining statistical significance. By applying Egger’s Test, we obtained an intercept beta coefficient of 4.54, with a 95% CI from −5.63 to 14.73 (*p* = 0.282). The intercept is located between –5.63 and 14.73, thereby indicating a low bias risk.

**Figure 5:**
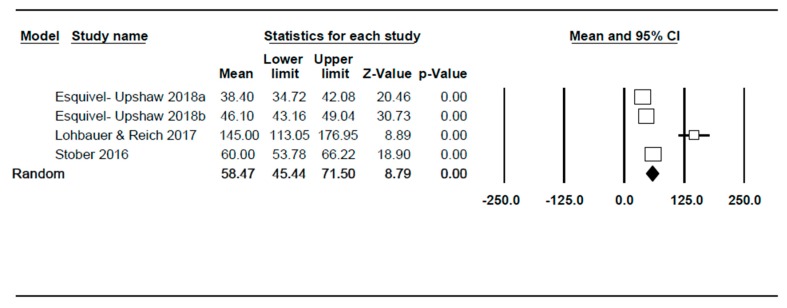
Forest-plot. Mean of the maximum wear reported by each author over time and the calculation of the global effect mean for monolithic zirconia restorations.

With regard to monolithic zirconia restorations, the studies included have estimated a wear corresponding to a range between 38.4 µm and 145 µm in a period spanning between 6 and 24 months (Figure 5). The combination of the random effect model studies has estimated a maximum wear of 58.47 µm, with a confidence interval at 95% (45.44–71.50). The meta-analysis has indicated high heterogeneity, with I^2^ = 95.9% and Q test = 72.5 (*p* < 0.001). The main source of heterogeneity is time as 6- to 24-month studies have been included.

**Figure 6:**
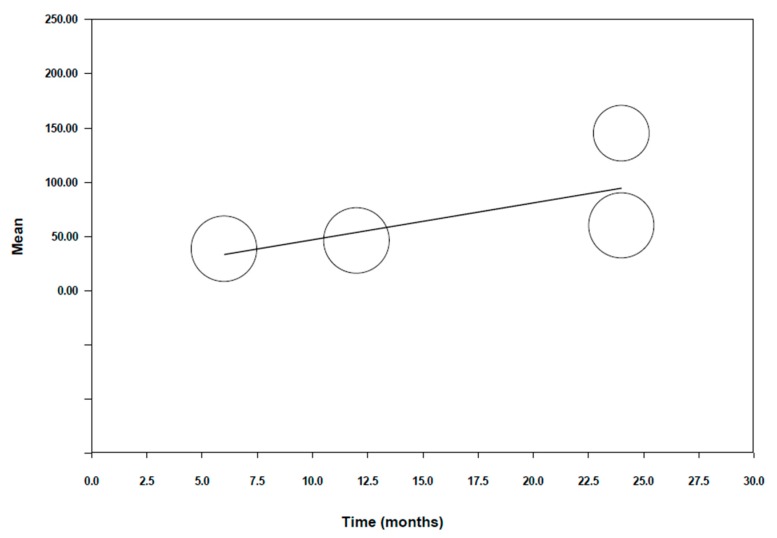
Graph depicting meta-regression for wear in monolithic zirconia vs. time.

To explain the high heterogeneity detected, in a similar procedure to the previous meta-analysis, a meta-regression was carried out with the variable time (Figure 6). A significant model was obtained; Q test = 3.73 (*p* = 0.053). However, the level of significance is not very high. The beta coefficient of the intercept was 12.92 with a CI at 95% (−48.22–74.06), giving a predictive capacity of 53% (R^2^ = 0.53); *p* value = 0.68. The beta coefficient of time is 3.40, with a *p* value = 0.053 and CI at 95% (−0.05–6.85). We can therefore conclude that in this case, time is a significant variable in the model (sustaining a maximum wear of 3.40 microns per month with respect to monolithic zirconia crowns), however not as much as in the case of wear in the antagonist tooth.

**Figure 7:**
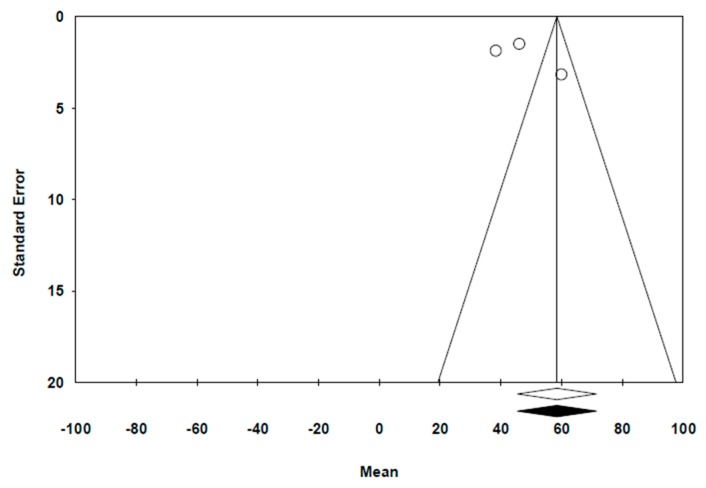
Funnel Plot with trim and fill (Duval and Tweedie).

The funnel plot (Figure 7) depicts a distribution for the results of the studies. By using the trim and fill method, the new estimation of effect was not different to the original measurement (58.47 µm). By using Egger’s Test, we obtained a beta coefficient for the intercept: 7.07, with a CI at 95%, ranging from −7.06 to 21.2 (*p* = 0.164). The mean is situated between −5.63 and 14.73, indicating a low bias risk.

## 4. Discussion

Significant wear is sustained over time both in monolithic zirconia crowns and in the antagonist tooth, however in the latter there is a greater degree of wear. In the meta-analysis a high degree of heterogeneity between the studies is observed, however the meta-regression test has shown that this heterogeneity is largely attributable to the time variable as it behaves as a significant variable in the model; therefore, we can affirm that over a greater period of time, greater wear takes place.

In our study we observe a mean maximum wear of the antagonist tooth to monolithic zirconia crowns of 95.45 µm (95% CI). Monolithic zirconia crowns suffer a mean maximum wear of 58.47 µm (95% CI).

The wear on enamel that is antagonist to monolithic zirconia was significantly greater than in the case of the natural tooth [7,14] but less than the wear in the antagonist enamel in ceramic–metallic crowns [7]. Stober appreciates a maximum vertical loss of antagonist enamel to metal–ceramic crowns of 151 ± 77μm [14] and Esquivel-Upshaw of 63 μm. [13]. The wear of the natural tooth was 75 ± 29 μm and 115 ± 60 μm [14] each.

In the study by Esquivel-Upshaw [13] there are no significant differences between the wear in natural enamel of the teeth and the wear of antagonist enamel in both monolithic zirconia restorations and in metal–ceramic ones. These researchers concluded that polished monolithic zirconia does not cause accelerated wear in the opposite enamel. Furthermore, this study showed that the wear in the enamel antagonist to zirconia was to a lesser degree than the wear sustained by ceramic–metallic crowns at 6 months but greater than the wear at 12 months. However, both results were not statistically significant. Further still, the study by Lohbauer and Reich [10] did not detect statistically significant differences between the antagonist enamel and zirconia restorations in terms of mean loss of volume and maximum vertical loss.

Among the factors that may influence the results of wear on the enamel from the said restorations, is the position itself of the restoration. According to the study by Mundhe [7], the wear on the natural enamel that is opposite to natural enamel, to metal–ceramic crowns, and to monolithic zirconia crowns in the premolar region was significantly less than in the molar region after 1 year. According to this study, this is attributable to thar fact that molars are subject to greater occlusal forces, given that the occlusion area, the number of contacts and the mastication forces in the molar region are greater than in the premolar region.

With regard to wear measurement, two methods are possible. The direct measurement method is the 3D intraoral scanning of the teeth, the technique in the study by Hartkamp [23]. The advantages of this measurement mode include enhanced precision and the simplification of the necessary steps [24]. However, the indirect technique in the evaluation of dental wear consists of making silicon impressions and then scanning the replicas made of plaster or resin [25]. This was the technique used in the majority of the studies that analyzed wear [7,9,10,13,14]; even though this method is precise and can quantify dental wear, the replication of the surface of the tooth and the manipulation of the 3D images could diminish the degree of precision [13], as is the case in the study by Lohbauer and Reich [10], where measurements of two samples were discarded due to the quality of the referential impression; the quality was inadequate to evaluate wear. We have not been able to make a quantitative comparison in meta-analysis regarding the measurement methodology, because only one study uses the direct measurement technique by intraoral scanning of the teeth [23] against five studies using the indirect technique through extraoral scanning of models [7,9,10,13,14], only one of which use a 3D noncontact profilometer [10].

The quantification of wear, in its definition, should reflect the loss of the tri-dimensional volume of dental tissue [26]. However, the majority of the articles [7,9,13,14,24] selected quantify wear solely on the basis of loss of height of the dental structure; as such, this measurement method can increase the discrepancies between the measurements of the various studies, as well as diminish the reliability of the results obtained.

We agree with Wulfman [27] on the lack of standardization to establish comparisons between the different clinical studies on dental and material wear and the inability to analyze an early wear of them due to the inaccuracy of these methods. We must seek the optimization of digital measurement protocols, avoiding accumulated measurement errors on replicas obtained from conventional prints. However, despite the impossibility of comparing objective wear data between the various work reviewed in our meta-analysis, we can affirm the influence of various aspects such as the surface treatment of restoration, intraoral position in which it is placed, the gender of the patient or their parafunctional habits in the wear of the material and the natural tooth.

Another factor to be considered is the treatment of restoration surface. Mundhe [7] and other researchers [10,13,22] only measured the wear in enamel that is antagonist to polished zirconia without glazing. Furthermore, Stober [14] evaluated the wear in enamel antagonist to glazed zirconia; however, no study had contemplated a control group to enable a comparison of the results of wear between polished monolithic zirconia and glazed zirconia. The preference for the surface finish was based, in the majority of studies, on literature-based findings where in vitro studies showed that glazed zirconia presented greater dental wear than polished zirconia [28]. A possible explanation is that glazed surfaces wear out more quickly and lead to patches of roughness in the unpolished ceramic matter below [11,15].

The wear of the teeth opposite to monolithic zirconia crowns was significantly less in women than in men [14]. In this study, the patient’s age did not affect differences in wear. Furthermore, the wear in enamel opposite to zirconia in patients with high nocturnal muscular activity (31–100 episodes in 5 h) tended to be greater than in patients with low nocturnal muscular activity, though no statistical significance was established.

Only four of the studies reviewed had control groups. Of these, two studies contemplated control as the wear between two natural teeth [7,14] while another two defined control as wear between the natural tooth and the tooth antagonist to ceramic–metallic crowns [7,13]. Due to the small number of studies analyzing a comparison of wear, such a comparison could not be made quantitatively in the meta-analysis.

The literature shows a mean value for natural wear of the teeth: 15 μm at 6 months [29] and 28 μm at 2 years [30]; these values are much less than the values of wear in the tooth that is antagonist to the monolithic zirconia restorations reported in this study. This can nonetheless be due to the studies included in the meta-analysis effectively convey the mean of maximum wear. However, these values of maximum wear are even lower than those reported for mean wear of the natural tooth in a patient with bruxism, producing a wear of 165 μm at 6 months [31].

## 5. Conclusions

Considering the limitations of the present study, it can be concluded that natural teeth antagonist to monolithic zirconia crowns are subject to significant wear over time which is greater than the wear sustained in the crowns themselves. This wear is influenced by the surface treatment of ZM crowns (glazed or polished), the position of the restorations (more common in molars than in pre-molars), gender (less frequent in women than in men) and the parafunctional habits of patients.

It is not possible to establish an objective and quantitative objection in relation to natural enamel wear or ceramic–metallic crowns. Further studies are needed with larger samples and longer follow-up periods, together with adequate control groups, if an in-depth analysis is to be obtained. 

## Figures and Tables

**Table 1:** Quality of the studies in the Newcastle–Ottawa scale for cohort studies.

AUTHOR (Year)	SELECTION				COMPARABILITY	OUTCOMES			TOTAL
	1	2	3	4	5-6	7	8	9	
Kitaoka et al. 2018 [19]	*	NA	*	*	NA	*	*	*	6

* NA: non applicable. Criteria: (1) Representativeness of the exposed cohort: truly representative (*) or somewhat representative (*). (2) Selection of the non-exposed cohort: drawn from the same community as the exposed cohort (*). (3) Ascertainment of exposure: secure record (e.g., surgical record) (*) or structured interview (*). (4) Demonstration that outcome of interest was not present at start of study: yes (*). (5–6) Comparability of cohorts on the basis of the design or analysis controlled for confounders: for the most important factor (*), for other factors (*). (7) Assessment of outcome: independent blind assessment or record linkage (*). (8) Was follow-up long enough for outcomes to occur (6 months) (*). (9) Adequacy of follow-up of cohorts: subjects lost to follow up unlikely to introduce bias – number lost less than or equal to 20% (*).

**Table 2:** Quality of the studies according to PEDro scale for clinical trials.

AUTHOR (Year)	Criteria											Total
	1	2	3	4	5	6	7	8	9	10	11	
Mundhe et al. (2015), [7]	Sí	Sí	Sí	Sí	No	No	No	Sí	Sí	Sí	Sí	8
Stober et al. (2016), [14],	Sí	No	No	Sí	No	No	No	Sí	Sí	Sí	Sí	6
Lohbauer et al. (2017), [10]	Sí	No	No	Sí	No	No	No	Sí	Sí	Sí	Sí	6
Hartkamp et al. (2017), [23]	Sí	No	No	Sí	No	No	No	Sí	Sí	Sí	Sí	6
Esquivel-Upshaw et al. (2018), [13]	Sí	Sí	Sí	Sí	Sí	No	No	Sí	Sí	Sí	Sí	9
Pathan et al. (2018), [9]	Sí	-	-	-	No	No	No	Sí	Sí	-	No	3
Tang et al. (2019), [3]	Sí	No	No	Sí	No	No	No	Sí	Sí	Sí	Sí	6

Criteria: (1) Eligibility criteria were specified. (2) Subjects were randomly allocated to groups. (3) Allocation was concealed. (4) The groups were similar at baseline regarding the most important prognostic indicators. (5) There was blinding of all subjects. (6) There was blinding of all therapists who administered the therapy. (7) There was blinding of all assessors who measured at least one key outcome. (8) Measures of at least one key outcome were obtained from more than 85% of the subjects initially allocated to groups. (9) All subjects for whom outcome measures were available received the treatment or control condition as allocated or, where this was not the case, data for at least one key outcome was analyzed by “intention to treat”. (10) The results of between-group statistical comparisons are reported for at least one key outcome. (11) The study provides both point measures and measures of variability for at least one key outcome.

**Table 3:** Mechanical complications. Monolithic zirconia crown units acting on the natural antagonist tooth.

Author Year	Title/Journal	Sample (*n*)	Follow-up Time (Months)	Mechanical Complications	
				Oclusal Wear	Other
**Mundhe et al.** **2015** **[7]**	Clinical study to evaluate the wear of natural enamel antagonist to zirconia and metal ceramic crowns. *J Prosthet Dent.*	10 patients with 10 monolithic zirconia crowns and 10 MC crowns.	12 months	Wear on natural enamel: - Control group (enamel–enamel): lowest wear (*p* < 0.001). - Experimental groups (enamel–MC and enamel–monolithic zirconia): higher wear than control group (*p* < 0.001), with enamel–MC wear being higher than enamel–zirconia wear (*p* < 0.001). - Wear in the premolar region was lower than in the molar region in all groups (*p* < 0.001).	-
**Stober et al.** **2016** **[14]**	Clinical assessment of enamel wear caused by monolithic zirconia crowns. *J Oral Rehabil.*	12 patients with 12 monolithic zirconia single crowns.	24 months	Mean vertical loss (± standard deviation): - CMonolithic zirconia crowns: 14 ± 5 μm. - Antagonist enamel: 46 ± 30 μm. - Control teeth enamel: 19 ± 9 μm and 26 ± 13 μm. Mean maximum vertical loss (± standard deviation): - Monolithic zirconia crowns: 60 ± 11 μm. - Antagonist enamel: 151 ± 77 μm. - Control teeth enamel: 75 ± 29 μm and 115 ± 60 μm. Statistical analysis revealed significant differences between enamel wear of zirconia-opposed teeth and control teeth (*p* = 0.01).	-
**Lohbauer and Reich** **2017** **[10]**	Antagonist wear of monolithic zirconia crowns after 2 years. *Clin Oral Investig.*	10 patients with 14 monolithic zirconia single crowns.	24 months	Mean volume loss (mm^3)^: - Antagonist enamel (*n* = 7): 0.361 ± 0.485 mm^3^. - Monolithic zirconia crowns (*n* = 10): 0.333 ± 0.267 mm^3^. Mean maximum vertical loss (mm): - Antagonist enamel: 0.204 ± 0.067 mm. - Monolithic zirconia crowns: 0.145 ± 0.061 mm. No significant difference was found between natural enamel antagonists and ceramic restorations (*p* > 0.05).	-
**Hartkamp et al.** **2017** **[23]**	Antagonist wear by polished zirconia crowns. *Int J Comput Dent*	9 patients with 13 monolithic zirconia single crowns.	24 months	Mean maximum vertical loss of monolithic zirconia antagonist enamel: 115 ± 71 μm.	-
**Kitaoka et al.** **2018** **[19]**	Clinical Evaluation of Monolithic Zirconia Crowns: A Short-Term Pilot Report. *Int J Prosthodont*	18 patients with 26 monolithic zirconia single crowns.	24 months	- Crack on the enamel of opposing teeth (*n* = 3; 10.34%). - Attrition on antagonist tooth: (*n* = 7; 24.14%).	- Marginal integrity, surface and anatomical form excellent or acceptable. - Unacceptable color (*n* = 1; 3.85%).
**Esquivel-Upshaw et al.** **2018** **[13]**	Randomized clinical study of wear of enamel antagonists against polished monolithic zirconia crowns. *J Dent*	25 patients with 16 monolithic zirconia crowns and 14 MC crowns.	12 months	Mean maximum vertical loss: - Monolithic zirconia antagonist enamel: 70.3 ± 4 μm. - Monolithic zirconia crown: 46.1 ± 6 μm. - MC antagonist enamel: 63 μm. - MC crown: 49.5 μm. No significant difference between wear of MC crown antagonist enamel and monolithic zirconia (*p* > 0.05).	-
**Pathan MS et al.** **2019** **[9]**	Assessment of antagonist enamel wear and clinical performance of full contour monolithic zirconia crowns one-year results of a prospective study. *J Prosthodont*	60 patients with 60 monolithic zirconia single crowns	12 months	- Mean wear of natural antagonist teeth: 16.3 μm.	- Marginal adjustment changes (*n* = 5.4, 9%). - Anatomical crown shape changes (*n* = 2, 3.3%). - Crown surface texture changes (*n* = 2, 3.3%). - Shade, color and crown translucency changes (*n* = 5, 8.3%).
**Tang Z et al.** **2019** **[3]**	Clinical evaluation of monolithic zirconia crowns for posterior teeth restorations. *Medicine*	46 patients with 49 monolithic zirconia single crowns	24 months	Antagonist tooth wear: - Grade 1 (only enamel wear and changes in occlusal surface morphology): *n* = 3 (6.12%). - Grade 2 (mild dentin wear, exposure of occlusal dentine with an area of ≤2 mm^2^, and decreased crown height due to morphological change in the occlusal surface): *n* = 3 (6.12%). - Crack (*n* = 1; 2.04%).	- Acceptable anatomical crown shape (*n* = 3; 6.12%). - Acceptable crown surface texture (*n* = 1; 2.04%).

**Table 4:** Measurement methods, surface treatment (finishing) and unit of measurement of results of the studies reviewed.

Author, Year	Measurement Methodology	Finishing	Outcomes
Mundhe et al. (2015) [7]	- Polyvinyl siloxane impressions before treatment and 1 year after cementation. - Casts: Type III gypsum. - Scanned using a 3D white light scanner (SmartSCAN^3D^ HE scanner; Breuckmann), (precision: ±9 µm). - Image superimposition (Polyworks, Innovmetric Software). - Wear at occlusal contact areas.	- Zirconia: polish (no glazing); before cementation. - Metal–ceramics: glaze.	- Mean vertical loss (µm).
Stober et al. (2016) [14]	- Polyvinyl siloxane impressions at baseline and at 6, 12, and 24 months. - Cast: type IV dental stone. - Scanned using a 3D laser scanner (Laserscan 3D and Match 3D, version 1.6; Willytec). - Accuracy: 10 µm. - Image superimposition. - Wear at occlusal contact areas.	- Zirconia: Glaze after polish during manufacture of crowns; polish after occlusal adjustment.	- Mean vertical loss (µm).Mean maximum vertical loss* (µm). - Mean maximum vertical loss* (µm).
Lohbauer et al. (2017) [10]	- A-silicone Flexitime impressions at baseline and 24 months after cementation. - Cast: epoxy resin material. - Scanned using 3D high-resolution noncontact profilometer (CT 100, Cybertechnologies) (lateral step size of 5 µm). - Image superimposition: software (Scan CT V8.4, Cybertechnologies, Ingolstadt). - Wear of regions-of-interest.	- Zirconia: polish (no glazing); polish after occlusal adjustment.	- Mean maximum vertical loss (mm). - Mean volume loss (mm^3^).
Hartkamp et al. (2017) [23]	- Intraoral digital scan at baseline and after 12 and 24 months. - Threshold: 30 µm. - Image superimposition. - Wear at occlusal contact areas.	- Zirconia: polish (no glazing).	- Mean maximum vertical loss (µm).
Esquivel et al. (2018) [13]	- Polyvinyl siloxane impressions at baseline and at 6 and 12 months after cementation. - Cast: white gypsum material - Scanned using a 3D laser scanner (CS2, Straumann, Alemania). - Accuracy: 20 µm. - Image superimposition. - Wear at occlusal contact areas.	- Zirconia: polish (no glazing). - Metal–ceramics: polish (no glazing).	- Mean maximum vertical loss (µm).
Pathan et al. (2019) [9]	- Addition silicone impressions at baseline and at 6 and 12 months after cementation. - Casts: vacuum-mixed die Stone. - Scanned using a 3D laser scanner (REXCAN CS+; Solutionix, Seoul, Korea). - Image superimposition: software (Geomagic Qualify; 3D Systems, Inc., Morrisville, NC, USA). - Threshold: −30 μm. - Wear at occlusal contact areas.	- Zirconia: Glaze after polish during manufacture of crowns; polish after occlusal adjustment.	- Mean vertical loss (µm).

* Mean Maximum Vertical Loss: a mean value of ten depth values around the maximum depth peak from each differential scan area on an investigated tooth.
